# A quasi-experimental study to estimate effectiveness of seasonal malaria chemoprevention in Aweil South County in Northern Bahr El Ghazal, South Sudan

**DOI:** 10.1186/s12936-024-04853-x

**Published:** 2024-01-24

**Authors:** Jamshed Khan, Maria Suau Sans, Francis Okot, Abubaker Rom Ayuiel, Jonathan Magoola, Christian Rassi, Sikai Huang, Denis Mubiru, Craig Bonnington, Kevin Baker, Julla Ahmed, Chuks Nnaji, Sol Richardson

**Affiliations:** 1 Malaria Consortium, Juba, South Sudan; 2 Malaria Consortium, London, UK; 3 Vanke School of Public Health, Tsinghua University, Beijing, China; 4 Department of Global Public Health, Karolinska Institute, Stockholm, Sweden; 5 Ministry of Health, Juba, South Sudan

**Keywords:** Seasonal malaria chemoprevention, Malaria, South Sudan, Sulfadoxine–pyrimethamine, Amodiaquine, Effectiveness

## Abstract

**Background:**

Seasonal malaria chemoprevention (SMC) is an effective intervention to prevent malaria in children in locations where the burden of malaria is high and transmission is seasonal. There is growing evidence suggesting that SMC with sulfadoxine–pyrimethamine and amodiaquine can retain its high level of effectiveness in East and Southern Africa despite resistance concerns. This study aims to generate evidence on the effectiveness of SMC when delivered under programmatic conditions in an area with an unknown anti-malarial drug resistance profile in the Northern Bahr el-Ghazal region of South Sudan.

**Methods:**

A non-randomized quasi experimental study was conducted to compare an intervention county with a control county. Five monthly SMC cycles were delivered between July and November 2022, targeting about 19,000 children 3–59 months old. Data were obtained from repeated cross-sectional household surveys of caregivers of children aged 3–59 months using cluster sampling. Wave 1 survey took place in both counties before SMC implementation; Waves 2 and 3 took place after the second and fourth monthly SMC cycles. Difference-in-differences analyses were performed by fitting logistic regression models with interactions between county and wave.

**Results:**

A total of 2760 children were sampled in the study across the three survey waves in both study counties. Children in the intervention arm had 70% lower odds of caregiver-reported fever relative to those in the control arm during the one-month period prior to Wave 2 (OR: 0.30, 95% CI 0.12–0.70, p = 0.003), and 37% lower odds in Wave 3 (OR: 0.63, 95% CI 0.22–1.59, p = 0.306) after controlling for baseline difference between counties in Wave 1. Odds of caregiver-reported RDT-confirmed malaria were 82% lower in the previous 1-month period prior to Wave 2 (OR: 0.18, 95% CI 0.07–0.49, p = 0.001) and Wave 3 (OR: 0.18, 95% CI 0.06–0.54, p = 0.003).

**Conclusion:**

These results show high effectiveness of SMC using SPAQ in terms of reducing malaria disease during the high transmission season in children 3–59 month. Despite the promising results, additional evidence and insights from chemoprevention efficacy cohort studies, and analyses of relevant resistance markers, are required to assess the suitability of SMC for this specific context.

**Supplementary Information:**

The online version contains supplementary material available at 10.1186/s12936-024-04853-x.

## Background

Seasonal malaria chemoprevention (SMC) with sulfoxide–pyrimethamine (SP) and amodiaquine (AQ) has been recommended since 2012 by the World Health Organization (WHO) as an effective strategy to prevent malaria among children below 5 years in the Sahel region of Africa [1]. SMC traditionally involves the administration of four monthly courses of SP + AQ to children between 3 and 59 months during the high-transmission season [2]. This intervention has been shown to be 88.2% effective at preventing clinical malaria in children under five during high-risk periods [3]. Since the WHO recommendation was made, implementation studies have been conducted in Sahelian and sub-Sahelian regions such as The Gambia, Chad, Burkina Faso, Mali and Senegal, which confirmed the effectiveness and cost-effectiveness of SMC in real-world conditions [3–9]. For those reasons, the scale-up of SMC in West and Central Africa is often seen as a success story. East and Southern African regions were not prioritized for SMC scale-up due to high levels of SP resistance. However, a recent study provided the first published evidence of effectiveness of SMC in a high resistance setting found the SMC using SP + AQ provided high level of protective effect (92%) against malaria during the peak transmission season in children aged 3–59 months in the Karamoja sub-region of Uganda [10].

South Sudan is among the countries with highest malaria transmission, with almost 3 million malaria cases estimated in 2021 [11]. Malaria is the leading cause of death in the country, and control efforts are challenged by the ongoing conflict situation, high levels of poverty and malnutrition [12, 13]. Malaria is endemic in all the regions, with prevalence peaking by the end of the rainy season from September to November. In the north of the country, total rainfall volumes are lower compared to the south, and tend to last between 5 and 6 months [14]. The country is also at one of the highest climate crisis risks, already suffering from increased flooding and changes in the rain seasonality, which is expected to worsen the malaria situation [15].

According to DHIS2 data and the Malaria Indicator Survey of South Sudan, Northern Bahr el Ghazal has one of the highest reported burden of malaria in the country for 3 to 59 months old children, with 32.5% of them testing positive based on malaria microscopy [14]. Over the recent years, the security context of Northern Bahr el Ghazal has been relatively calm, allowing for development support organizations to establish themselves in its capital, Aweil Town. The seasonal pattern of malaria transmission and disease burden in the area, coinciding with the peak of the rainy season, meets the current SMC eligibility criterion which requires that at least 60% of cases occur during a maximum of 4 months (Additional file 1: Fig. S1).

In recognition of the need to prevent malaria and reduce morbidity and mortality among children, and the potential benefits of SMC in achieving those goals, SMC has been included in South Sudan’s current national malaria strategy [16]. The Malaria Consortium, one of the leading organizations implementing SMC worldwide, has a well-established field operations coordination office in Northern Bahr el Ghazal, from where it implements several health-related programmes in partnership with the National and State Ministries of Health. It is acknowledged that, an intervention’s success and impact vary across settings, and implementation programmes need to be context specific. Therefore, the Malaria Consortium in partnership with the National Malaria Control Programme (NMCP) of South Sudan, developed a multi-component implementation research study exploring the effectiveness, feasibility, and acceptability of SMC, as well as the chemoprevention efficacy of SPAQ in Aweil South County, Northern Bahr el Ghazal state. SMC was implemented by *Médecins Sans Frontières* in the Aweil County of Northern Bahr el Gazhal during the 2021 and 2022 high malaria incidence seasons, based on a fixed-point and a door-to-door delivery approach, respectively. However, impact outcomes of the intervention are not publicly available [17].

This paper presents the results of the effectiveness component of the implementation research, the aim of which was to investigate the effectiveness of SMC, on an intention-to-treat basis, for prevention of clinically-significant cases of malaria among children aged 3–59 months.

## Methods

### Study design

This study was a component of a Type II hybrid effectiveness-implementation study design [18]. It involved a non-randomized controlled study based on a cluster sampling approach with repeated cross-sectional surveys of caregivers of children aged 3–59 months.

### Study settings

**Fig. 1:**
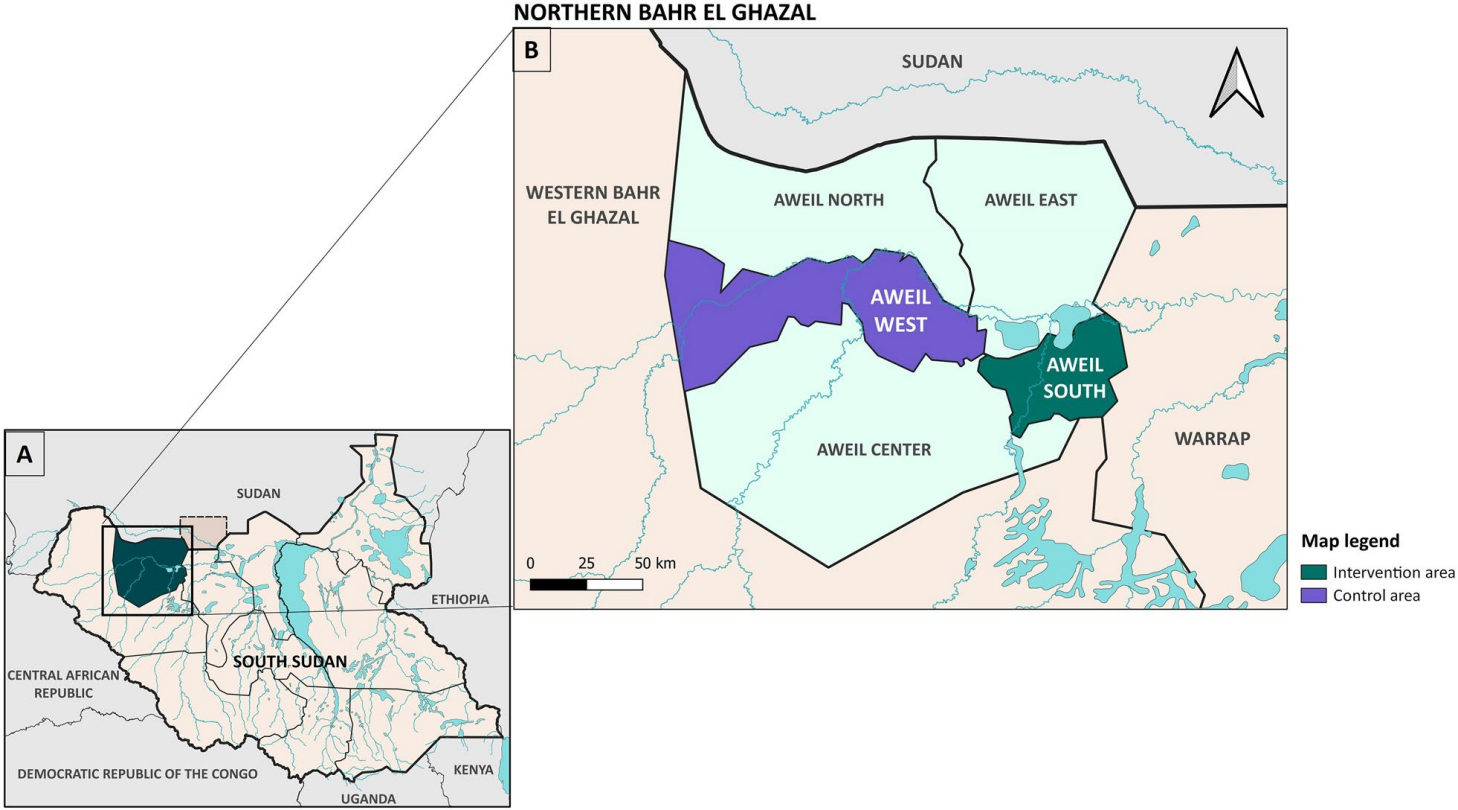
Maps showing the Northern Bahr el-Ghazal region in South Sudan (**A**) and the study counties (**B**)

Northern Bahr el Ghazal state is part of Greater Bahr el Ghazal and includes five counties: Aweil South, East, West, North and Center with a total area of 30,543 km (Fig. 1A, B). Aweil Town is the capital of the state, and most of the state’s population is composed of Dinka and Jurchol tribe members, and a minority of Luo tribe members, whose main source of livelihood is agriculture and livestock. Floods occur annually from June to November and hinder routine life, causing internal displacements.

### Seasonal malaria chemoprevention implementation

National, state, county and boma stakeholders’ meetings were organized before SMC implementation. A total of 288 community health workers, nationally known as Boma Health Workers (BHWs), and 18 BHW supervisors, were trained for this intervention. BHWs delivered SMC house-to-house to eligible children in the intervention county (Fig. 1) and directly observed treatment for the first doses of SP + AQ. Instructions on how to administer day 2 and 3 AQ doses were given to caregivers. Eligibility criteria for SMC administration included: children aged 3–59 months at the time of SMC distribution and residing in the intervention area, afebrile with no other malaria-associated symptoms in the 48 h prior to recruitment. Children with known allergies or adverse reactions to SP or AQ, that received a sulfa-based medication for treatment or prophylaxis, receiving azithromycin, treated with AQ in the past 28 days, suffering from severe malnutrition or HIV positive were deemed ineligible.

Before the start of each SPAQ distribution cycle, sensitization activities including radio announcements and community engagement meetings with community chiefs, religious leaders and caregivers were conducted. Each study and SMC cycle was monitored and supervised by Malaria Consortium staff and SMOH/NMOH staff.

Based on administrative data collected by BHWs, cycle 1 of SPAQ administration was conducted from 22nd to 25th July and reached 17,681 children in the intervention county, Aweil South. In Cycle 2, SPAQ distribution was conducted from the 19th to the 22nd August and reached 17,041 children. In Cycle 3, distribution was conducted from the 17th to the 20th September and reached 17,007 children. In Cycle 4, distribution was conducted from the 17th to 20th October and reached 17,624 children. Cycle 5 of SPAQ administration was conducted between the 17th and the 20th of November and reached 17,532 children.

### Survey and data collection methods

The impact of SMC on caregiver-reported fever among eligible children, and caregiver-reported RDT-confirmed malaria was investigated using difference-in-differences (DiD) analyses for prevention of clinically-significant cases of malaria among children aged 3–59 months on an intention to treat basis. DiD is a quasi-experimental approach that compares the changes in outcomes over time between control and intervention groups [19]. The study team did not perform RDT confirmation themselves, RDT-confirmed malaria cases were reported by caregivers in the cross-sectional surveys, such RDTs would have been conducted by the BHWs or at the health facilities to confirm malaria cases.

Data for these analyses were collected through three cross–sectional surveys. The first, wave 1, took place in July 2022 and questions on malaria outcomes covered the period immediately prior to the start of the SMC round in Aweil South and the rainy season (7 June to 7 July). Wave 2 and Wave 3 cross-sectional surveys took place in September and November 2022 and covered the periods corresponding to the one-month period following the start of delivery of SMC in cycle 2 (18 August to 17 September) and cycle 4 (17 October to 16 November), respectively.

The sample size for each survey was calculated to give 80% statistical power to detect at the 5% significance confidence level using a two-tailed test, a 40% or more difference in malaria incidence between implementation and control areas. All 23 Bomas in the intervention county (Aweil South) were included in the survey and 20 households were randomly selected from each, resulting in a sample size of 460 households selected for the intervention arm. Due to the lack of available data on the Boma populations at the time of study planning, in the control area (Aweil West), a simple random sample of 23 were selected from the total of 31 Bomas, with 20 households selected from each. These clusters were re-randomized for each wave. The overall target sample size for each wave was 920 households. As Bomas were selected using a simple random approach, post-hoc survey weights were generated based on their population size to ensure representativeness of analysis results.

Surveys were administered by pairs of data collectors, with responses recorded on mobile devices using the application SurveyCTO (version 2.80) [20]. In each randomly selected household, a roster of all children aged 3–119 months was taken and one child aged 3–59 months was selected at random with the aid of SurveyCTO, as described elsewhere [21]. Surveys forms were in English and translated in situ to respondents in their language. The survey contained a range of questions on child, caregiver and household-level variables. Before participating in the survey, caregivers and heads of household provided written informed consent.

### Outcome measures

The main outcomes of this study were: caregiver-reported fever among eligible children, and caregiver-reported HRP-2 RDT-confirmed malaria in the relevant month period covered by each survey wave. Secondary outcomes included SMC coverage in terms of the proportion of children in the intervention area who received Day 1 SPAQ and the proportion of those who received the full three-day course of SPAQ across monthly cycles.

### Statistical analysis

The weighted proportions of children who experienced each of the two outcomes were estimated in each wave by county with 95% confidence intervals (95% CI). A descriptive analysis of the sample recorded in each wave was conducted, with the following categorical variables: caregiver-reported fever in the previous 1-month period; caregiver-reported RDT-confirmed malaria in the previous 1-month period; child’s sex; child’s age; caregiver’s sex; caregiver’s age; caregiver’s partnership status; caregiver’s self-reported literacy; caregiver’s level of education; caregiver’s occupation; and household ownership of a mosquito net. Indoor residual spraying was excluded from the analysis as no campaigns had been delivered in the intervention or control districts within the past 12 months prior to the SMC round. Covariate balancing between the intervention and control counties was investigated in each wave, and between waves, using weighted Chi-square tests.

DiD analysis was performed by fitting logistic regression models with interactions between county and wave. This facilitated a comparison of the change in odds of fever and RDT-confirmed malaria from the pre-intervention period (Wave 1) to the period of the SMC round (Wave 2 and Wave 3) between the intervention and control counties to give estimates of effect of SMC in each wave expressed as odds ratios (OR) with 95% CIs.

For each of the two outcomes, three models were fitted: Model 1 was the ‘unadjusted’ model without additional covariates beyond county, survey Wave and interaction terms; Model 2 further adjusted for the child’s age and sex; Model 3 represented the ‘full’ model including all variables selected for inclusion from the full list following the forward stepwise procedure by sequentially adding terms with likelihood-ratio p < 0.1 and removing those with p ≥ 0.2. For both outcomes, only household net ownership and the housing quality scale met the criteria for inclusion. Variables considered for inclusion in Model 3 comprised all those mentioned above, in addition to a housing quality index was defined using Mokken scale analysis, to be described in a future publication. Survey weights were applied to all models [22]. STATA 17 software was used to conduct the analysis of this study [23].

### Sensitivity analysis

The primary analysis was conducted on an intention to treat basis. In addition, sensitivity analyses were conducted excluding children in the intervention county who did not receive Day 1 SPAQ in the month before the survey and those in the control county who received Day 1 SPAQ in the month before the survey. A second sensitivity analysis further excluded those children in the intervention county who did not receive AQ on Day 2 and Day 3.

### Ethical considerations

This study received ethics approval by the ethical review board at the Republic of South Sudan Ministry of Health in Juba.

## Results

### Participants’ characteristics

A total of 2760 children aged 3–59 months were enrolled in the study across the three survey waves (946 in Wave 1, 902 in Wave 2 and 912 in Wave 3) in both study counties. Tables 1 and 2 present participants’ child-, caregiver- and household-level characteristics at baseline and across the three survey waves. Overall, there were no significant differences observed in the characteristics when compared across survey waves, with the exception of household net ownership (p = 0.011) and sex (p = 0.023). Similarly, after sample weighting to account for survey design, there was balance in the participants’ characteristics between the intervention and control counties within each survey wave, except for household net ownership, which was significantly higher in the control county (p < 0.0001). Table 2 shows the characteristics of the analytic sample, including child, caregiver, and household variables, by county for the baseline survey, while those of Wave 2 and Wave 3 surveys are presented in Additional file 1: Table S1 and S2.

**Table 1:** Participants’ characteristics across survey waves in both Aweil South and Aweil West counties, June–November 2022

Variable		Category	Wave 1 (n = 946)			Wave 2 (n = 902)			Wave 3 (n = 912)			p^#^
			n	%	Weighted %	n	%	Weighted %	n	%	Weighted %	
Child-level factors	Fever	Yes	699	73.9	71.5	677	75.1	75.7	653	71.6	73.0	0.689
		No	247	26.1	28.6	225	24.9	24.3	259	28.4	27.1	
	RDT-confirmed malaria	Yes	473	50.0	46.9	532	59.0	60.0	534	58.6	58.5	0.069
		No	473	50.0	53.1	370	41.0	40.0	378	41.4	41.5	
	Sex	Male	450	47.6	46.9	456	50.6	52.6	443	48.6	48.0	**0.023**
		Female	496	52.4	53.1	446	49.4	47.4	469	51.4	52.0	
	Age	3–12 months	113	11.9	8.6	83	9.2	9.6	46	5.0	6.8	0.320
		1 year	209	22.1	23.3	134	14.9	15.2	189	20.7	20.2	
		2 years	175	18.5	20.9	190	21.1	20.8	225	24.7	23.6	
		3 years	237	25.1	24.0	219	24.3	22.7	230	25.2	22.4	
		4 years	149	15.8	15.9	196	21.7	21.3	169	18.5	19.7	
		5 years	63	6.7	7.4	80	8.9	10.5	53	5.8	7.4	
Caregiver-level factors	Age	Under 20 years	151	16.0	11.1	123	13.6	16.5	159	17.4	18.7	0.184
		20–29 years	345	36.5	37.1	360	39.9	39.1	413	45.3	43.6	
		30–39 years	299	31.6	36.0	312	34.6	32.3	244	26.8	26.8	
		40–49 years	113	11.9	11.3	87	9.6	10.0	66	7.2	7.5	
		50 or more above	38	4.0	4.5	20	2.2	2.2	30	3.3	3.5	
	Sex	Male	230	24.3	21.7	213	23.6	22.9	204	22.4	23.8	0.802
		Female	716	75.7	78.3	689	76.4	77.1	708	77.6	76.2	
	Partnership status	Married/partnered	856	90.5	93.4	821	91.0	88.0	803	88.0	85.3	0.093
		Non-partnered	90	9.5	6.7	81	9.0	12.0	109	12.0	14.7	
	Literacy	Yes	385	40.7	41.2	452	50.1	48.5	454	49.8	47.0	0.497
		No	561	59.3	58.8	450	49.9	51.5	458	50.2	53.0	
	Education	None	558	59.0	57.3	489	54.2	52.5	503	55.2	53.5	0.869
		Informal	150	15.9	15.5	156	17.3	16.6	171	18.8	17.7	
		Primary or above	238	25.2	27.2	257	28.5	30.9	238	26.1	28.8	
	Occupation	Non-employed	136	14.4	13.7	133	14.7	12.7	142	15.6	16.1	0.252
		Unemployed	23	2.4	2.8	66	7.3	8.1	76	8.3	8.2	
		Agricultural	705	74.5	75.9	625	69.3	71.7	631	69.2	68.1	
		Unskilled manual work	44	4.7	3.9	35	3.9	4.2	24	2.6	2.1	
		Skilled/Service/professional	38	4.0	3.8	43	4.8	3.4	39	4.3	5.5	
Household	Net ownership	Yes	504	53.3	51.6	632	70.1	69.8	685	75.1	73.3	**0.011**
		No	442	46.7	48.4	270	29.9	30.2	227	24.9	26.7	

Bold values indicate that the strength of evidence is significant at p<0.05

^#^χ^2^ test for comparison of proportions considering survey design

**Table 2:** Comparison of participants’ characteristics at baseline between Aweil South and Aweil West counties, June 2022.

Variable		Category	Aweil South (intervention)			Aweil West (control)			
			n	%	Weighted %	n	%	Weighted %	p^#^
Child	Fever	Yes	289	62.4	58.5	410	84.9	84.0	**0.007**
		No	174	37.6	41.5	73	15.1	16.0	
	RDT-confirmed malaria	Yes	218	47.1	48.0	255	52.8	45.7	0.808
		No	245	52.9	52.0	228	47.2	54.3	
	Sex	Male	221	47.7	46.3	229	47.4	47.5	0.733
		Female	242	52.3	53.8	254	52.6	52.5	
	Age	3–12 months	43	9.3	8.4	70	14.5	8.8	0.703
		1 year	107	23.1	22.0	102	21.1	24.6	
		2 years	83	17.9	19.7	92	19.0	22.0	
		3 years	129	27.9	27.5	108	22.4	20.6	
		4 years	68	14.7	13.4	81	16.8	18.2	
		5 years	33	7.1	8.9	30	6.2	5.9	
Caregiver	Age	Under 20 years	89	19.2	13.9	62	12.8	8.5	0.118
		20–29 years	150	32.4	30.3	195	40.4	43.7	
		30–39 years	158	34.1	40.2	141	29.2	31.9	
		40–49 years	47	10.2	10.8	66	13.7	11.8	
		50 or more above	19	4.1	4.8	19	3.9	4.2	
	Sex	Male	138	29.8	27.7	92	19.0	15.9	**0.025**
		Female	325	70.2	72.3	391	81.0	84.1	
	Partnership status	Married/partnered	432	93.3	95.3	424	87.8	91.4	0.155
		Non-partnered	31	6.7	4.7	59	12.2	8.6	
	Literacy	Yes	174	37.6	39.2	211	43.7	43.2	0.680
		No	289	62.4	60.8	272	56.3	56.8	
	Education	None	313	67.6	67.3	245	50.7	47.7	**0.042**
		Informal	82	17.7	18.7	68	14.1	12.4	
		Primary or above	68	14.7	14.1	170	35.2	39.9	
	Occupation	Non-employed	79	17.1	12.0	57	11.8	15.4	0.252
		Unemployed	13	2.8	1.9	10	2.1	3.6	
		Agricultural	352	76.0	82.7	353	73.1	69.3	
		Unskilled manual work	4	0.9	0.6	40	8.3	7.1	
		Skilled/Service/professional	15	3.2	2.9	23	4.8	4.6	
Household	Net ownership	Yes	165	35.6	30.3	339	70.2	72.3	**< 0.000**
		No	298	64.4	69.7	144	29.8	27.7	

Bold values indicate that the strength of evidence is significant at p<0.05

^#^χ^2^ test for comparison of proportions considering survey design

### SMC coverage

In the intervention county, Aweil South, caregiver-reported SMC coverage of Day 1 SPAQ was 92.0% (95% CI 83.8–95.8) in the second SMC cycle, and 93.8% (95% CI 85.3–97.5) in the fourth cycle (Additional file 1: Table S3). Among children who received Day 1 SPAQ, reported coverage of receipt of the full three-day course (that is, receiving both Day 2 and Day 3 AQ from caregivers in addition to receiving Day 1 SPAQ) was 88.2% (95% CI 77.2–94.3) and 99.2% (95% CI 97.3–99.7) in the second and fourth cycles, respectively.

In the control county, caregivers reported 23.6% (95% CI 9.6–47.4) of children received at least one dose of SMC medicines in the second cycle, and 14.1% (95% CI 0.5.6–31.6) in the fourth cycle.

### Distribution of fever and RDT-confirmed malaria across survey waves and counties

**Fig.2:**
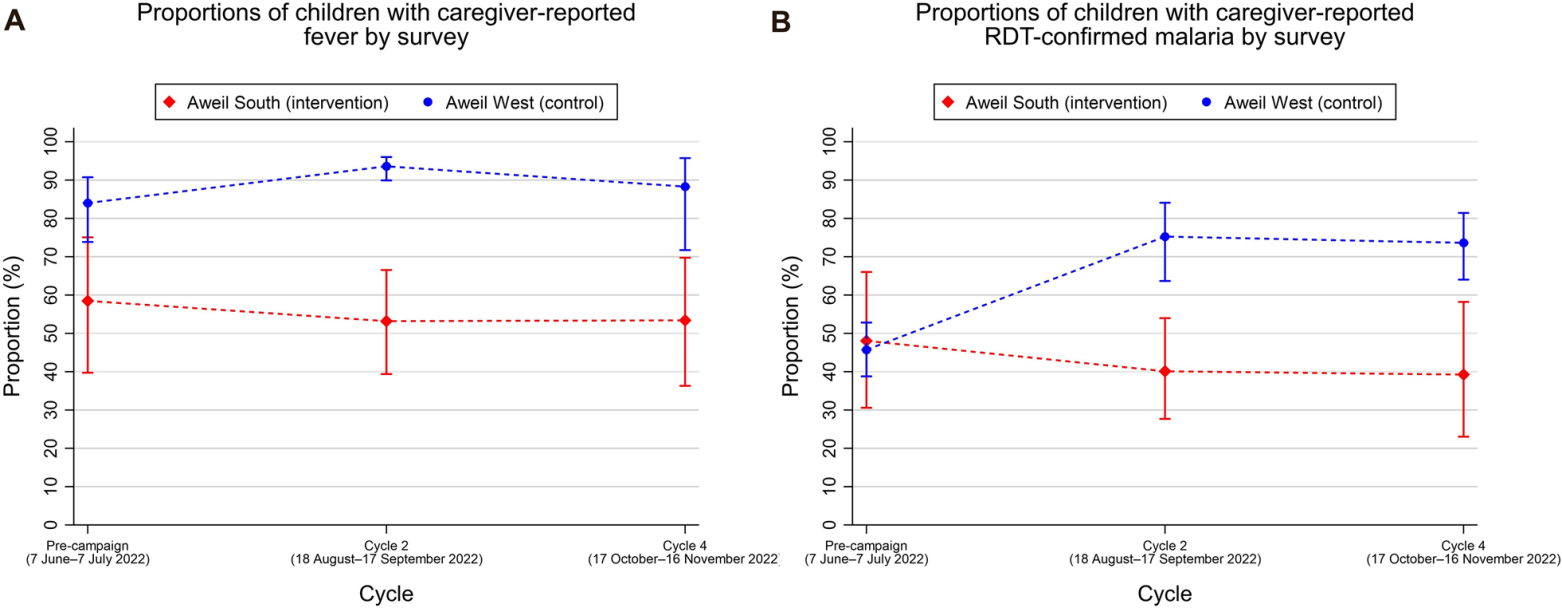
Proportions of children with caregiver-reported outcome. Weighted proportions of children with caregiver-reported fever (**A**) and caregiver-reported RDT-confirmed malaria (**B**) by county (study arm) and across study periods

Figure 2A shows the proportions of children in the intervention and control counties whose caregivers reported fever episodes during the month before the survey wave. For both outcomes, a decrease was observed following the second and fourth SMC cycles compared with the pre-SMC period in the intervention county and increased in the control county. In the intervention county, fever was reported in 58.52% (95% CI 40.12–75.62), 52.80% (95% CI 39.92–67.73) and 53.14% (95% CI 37.32–69.86) of children in the first, second and third survey waves, respectively. Among children in the control county, fever was reported in 84.9% (95% CI 73.42–91.26), 93.8% (95% CI 90.14–96.33) and 88.3% (95% CI 72.24–96.46) across the first to third survey waves.

Figure 2B illustrates the proportions of children in the intervention and control counties by survey wave whose caregivers reported they had experienced episodes of RDT-confirmed malaria in the one-month prior to the survey. In the first, second (following the second cycle) and third (following the fourth cycle) survey waves, 48.04% (95% CI 31.32–67.61), 40.52% (95% CI 28.23–54.04) and 38.8% (95% CI 23.32–58.11) of children had RDT-confirmed malaria, respectively. Among children in the control county, 52.81% (95% CI 39.12–53.43), 75.35% (95% CI 63.74–85.15) and 73.61% (95% CI 63.20–81.68) had malaria in the first, second and third survey waves.

### Estimates of protective effectiveness of SMC

Table 3 shows the results of DiD analyses for SMC effectiveness in terms of caregiver-reported fever and RDT-confirmed malaria. The OR for county shows the difference in odds of each outcome at baseline between counties, while the coefficients for Wave show the difference in odds of each outcome in each survey wave compared with the baseline survey (Wave 1) for both counties combined under the parallel trends assumption. The effect of SMC on the odds of reported malaria outcomes for each wave is shown by the interaction terms.

**Table 3:** Results of regression models for associations between SMC and caregiver-reported malaria outcomes among children aged 3–59 months using difference-in-differences analysis comparing Aweil South and Aweil West counties, June–November 2022

Outcome	Model description	Variable	Category	Odds ratio	95% CI	*p*
Caregiver-reported fever outcomes	Model 1: unadjusted model	County	Aweil South (intervention)	0.27	0.10–0.70	**0.008**
		Wave	Wave 2	2.78	1.77–4.37	**< 0.001**
			Wave 3	1.44	0.77–2.67	0.246
		Interaction	County*Wave 2	0.29	0.12–0.70	**0.007**
			County*Wave 3	0.56	0.20–1.54	0.252
	Model 2: adjusted for child age and sex	County	Aweil South (intervention)	0.26	0.11–0.62	**0.004**
		Wave	Wave 2	2.60	1.68–4.04	**< 0.001**
			Wave 3	1.39	0.72–2.66	0.315
		Interaction	County*Wave 2	0.29	0.12–0.70	**0.007**
			County*Wave 3	0.59	0.22–1.59	0.285
	Model 3: full model	County	Aweil South (intervention)	0.18	0.11–0.62	**< 0.001**
		Wave	Wave 2	2.40	1.68–4.04	**0.001**
			Wave 3	1.36	0.72–2.66	0.260
		Interaction	County*Wave 2	0.30	0.12–0.70	**0.003**
			County*Wave 3	0.63	0.22–1.59	0.306
Caregiver-reported RDT-confirmed malaria outcomes	Model 1: unadjusted model	County	Aweil South (intervention)	1.10	0.51–2.23	0.807
		Wave	Wave 2	3.61	1.93–6.77	**< 0.001**
			Wave 3	3.32	1.91–5.76	**< 0.001**
		Interaction	County*Wave 2	0.21	0.08–0.55	**0.003**
			County*Wave 3	0.21	0.06–0.62	**0.006**
	Model 2: adjusted for child age and sex	County	Aweil South (intervention)	1.10	0.51–2.38	0.803
		Wave	Wave 2	3.61	2.03–6.43	**< 0.001**
			Wave 3	3.31	1.91–5.73	**< 0.001**
		Interaction	County*Wave 2	0.20	0.08–0.54	**0.002**
			County*Wave 3	0.21	0.07–0.63	**0.006**
	Model 3: full model	County	Aweil South (intervention)	0.94	0.45–1.93	0.860
		Wave	Wave 2	3.39	1.79–6.43	**< 0.001**
			Wave 3	3.39	1.89–6.08	**< 0.001**
		Interaction	County*Wave 2	0.18	0.07–0.49	**0.001**
			County*Wave 3	0.18	0.06–0.54	**0.003**

Bold values indicate that the strength of evidence is significant at p<0.05

Results from the full model show that in the intervention county, the odds of caregiver-reported fever during the month prior to SMC implementation (Wave 1 survey) were 82% lower (OR: 0.18, 95% CI 0.11–0.62, p < 0.001) compared with the control county. The model shows a statistically-significant 70% reduction in odds of caregiver-reported fever in the intervention county relative to the control county during the one-month period prior to Wave 2 survey (OR: 0.30, 95% CI 0.12–0.70, p = 0.003), but a non-significant 37% reduction in the one-month period preceding Wave 3 survey (OR: 0.63, 95% CI 0.22–1.59, p = 0.306).

For RDT-confirmed malaria, there was no evidence to suggest that there was a difference in odds of caregiver-reported RDT-confirmed malaria between the intervention and control counties in the one-month period before SMC implementation (OR: 0.94, 95% CI 0.45–1.93, p = 0.860). Children in the intervention county had significantly lower odds of caregiver-reported RDT-confirmed malaria episodes in the previous one-month period prior to Wave 2 (OR: 0.18, 95% CI 0.07–0.49, p = 0.001) and Wave 3 (OR: 0.18, 95% CI 0.06–0.54, p = 0.003). Overall, these results indicate that SMC was associated with 82% lower odds of caregiver-reported RDT-confirmed malaria episodes during the previous one-month period among children in the intervention county, when compared with those in the control county.

### Sensitivity analysis

The results of the sensitivity analysis are shown in Additional file 1: Tables S4 and S5. They indicate that, after exclusion of children who did not follow the intended protocol for their county in terms of SMC treatment, the estimated effect sizes for SMC on the two outcomes were similar to those of the primary analysis.

## Discussion

### Summary of key findings

This study found that SMC delivered to age-eligible children under programmatic conditions was associated with 82% lower odds of caregiver-reported RDT-confirmed malaria episodes in the Aweil South County in the Northern Bahr El Ghazal region of South Sudan. These findings are consistent with, and add to, the growing body of evidence on the high level of SMC effectiveness reported in previous randomized and observational studies [3, 8, 24]. The study also found a reduction in the odds of caregiver-reported fever in children residing in the intervention county, relative to those in the control county. Discrepancy between the statistical significance of the two outcomes in wave 3 could be due to the reliability of the outcome as an indicator of malaria infection, with caregiver-reported RDT-confirmed malaria cases being more reliable, and the associated model outcomes more relevant for the interpretation of the study results.

More importantly, the study contributes to the emerging evidence that SMC deployed in new geographies outside of the Sahel region of West and Central Africa can retain its high level of protection against clinical malaria observed in Sahelian contexts [10].

### Implications of study findings

The deployment of SMC has traditionally been considered suitable in areas where parasite resistance to SMC medicines is low. This is recognition that resistance to SP or AQ may reduce the efficacy and effectiveness of SMC in protecting children against clinical malaria. For instance, there are concerns that SP efficacy may be undermined by drug resistance due to mutations in the dihydrofolate reductase (*dhfr)* and dihydropteroate synthetase (*dhps)* genes [25]. However, the relationship between the degree of resistance and the effectiveness of SMC has not yet been fully understood. It is hoped that SP may retain its effectiveness even in areas where resistance is high, as reflected in the updated WHO guidelines, which no longer define any geographical restrictions for the deployment of SMC in new geographies [26].

South Sudan faces ongoing humanitarian challenges, worsened by conflict and environmental challenges such as drought and flooding, of which impact on people’s livelihoods and access to education and water, sanitation and hygiene and health services [27]. As such, SMC implementation in the Aweil South County in the Northern Bahr El Ghazal region was affected by many of these contextual constraints like flooding, which also affected the control area (Aweil West). An effective delivery of SMC requires high levels of SMC coverage among the target population month-to-month. Achieving this in study context and similar settings requires adequate planning, resources, and integration into existing systems to ensure optimal uptake and coverage. Despite the operational challenges of delivering SMC in the study setting, a high level of SMC coverage was achieved, with over 90% of children receiving at least one dose of SMC across cycles. This indicates that door-to-door distribution of SMC medicines by BHWs was successful in reaching most of the target population of age-eligible children in the intervention county despite operational constraints. Similarly, caregiver reported adherence to unsupervised Day 2 and Day 3 AQ doses was also high. Findings also support current evidence on the feasibility of deploying SMC as a malaria prevention and control strategy in post-conflict settings with high burden of malaria [28]. Results from another component of Malaria Consortium’s broader implementation research which assessed feasibility and acceptability of SMC in the intervention setting will contribute additional evidence in this regard and published elsewhere.

Despite the promising effectiveness results seen in the current study, there is a need for further evidence to facilitate a more accurate understanding of the effectiveness and suitability of SMC, and to inform any decision on the continued implementation or scale-up of SMC in South Sudan and similar high resistance settings. For example, evidence from chemoprevention efficacy cohort studies and analyses of relevant resistance markers will enable further understanding of how well current SMC drugs work in curing existing infections and preventing new ones, how long they are likely to remain efficacious for, and if there’s a risk of negatively affecting other chemoprevention strategies which also use SP, such as intermittent preventive treatment in pregnancy and perennial malaria chemoprevention. Results anticipated from parallelly conducted resistance markers, chemoprevention efficacy and qualitative study components of the broader implementation research will make important contributions to that understanding. Specifically, they will improve current understanding of the role of drug resistance in determining SMC effectiveness, efficacy and potential scalability in South Sudan and similar locations in East and Southern African regions with high and heterogeneous SPAQ parasite resistant profiles. Moreover, ongoing monitoring and surveillance of SPAQ chemoprevention efficacy and associated resistance markers over time will be required in all locations where SMC may be deployed in the future.

### Study strengths, limitations, and implications for future research

While this study did not involve the random assignment of children to study arms, the DiD approach allowed the estimation of effect of SMC in the absence of randomization. As SMC was delivered in only one county and it was considered infeasible to conduct a randomized trial, either at the individual level or a cluster-randomized trial at the village level. The DiD method helps to account for significant differences in outcomes between the treatment and control groups, while also accounting for differences in those outcomes across the pre-treatment and post-treatment periods. A limitation of the DiD approach utilized here is that the parallel trends assumptions in the pre-implementation period were based on routine malaria surveillance data in both study counties, which may be unreliable due to data quality issues.

Another strength of this study was the intention-to-treat analysis approach. This enabled the measurement of the real-world effect of SMC, particularly as not all children in the intervention county received SMC medicines as intended from caregiver reports, while some children in the country county might have received SMC medicines. Although those reports could not be verified, nor was the extent to which there was contamination between study arms be ascertained, such occurrences are not uncommon in studies assessing the effect of public health interventions, particularly when delivered under programmatic conditions [29]. As a possible explanation for the higher-than-expected report of receipt of SMC medicines by children in the control county, it is hypothesized that some caregivers might not have understood the question, and responded ‘yes’ when asked if their children received SMC, mistaking SMC with other malaria medicines, or drugs such as Vitamin A or anthelminthics given to their children during health facility visits or health campaigns.

An important limitation of this study was its reliance on self-reporting by caregivers on the two outcome measures (fever and RDT-confirmed malaria cases) in addition to the caregiver- and household-level covariates tested for inclusion in the models. This may have introduced the risk of recall bias in the study. For some of these variables, responses may have been subject to social desirability bias. While the analyses adjusted for important covariates and potential confounders such as household net ownership and housing quality, results could have been influenced by effects of unmeasured confounders like health promotion campaigns and other interventions that might have influenced the outcomes of interest. However, there was no report on these in neither of the two study counties during the study period. Besides, the balance in child, caregiver, and household-level characteristics between the two counties should have minimized the impact of any unaccounted confounding factors.

This study was unable to measure the effect of SMC on the prevention of malaria infection, case severity and mortality. Another limitation regarding fever outcomes stemmed from the study’s inability to ascertain if reported fever cases by caregivers were indeed attributable to *Plasmodium* *falciparum* infection (rather than another cause, with coincidental malaria parasitaemia and RDT positivity). These are therefore empirical opportunities for future research efforts to address, part of which is the focus of other components of our SMC implementation research aiming to assess the chemoprevention efficacy of SMC in terms of clearing existing *P*. *falciparum* infections and preventing new ones in settings of high SPAQ resistance.

Surveys were conducted in series, with data collection in the intervention county taking place a week after the control county. This may have biased the effectiveness estimates, as caregivers in the intervention county were asked to report on malaria-related outcomes with a longer time lag between the end of the period referred to in the questionnaire and the date of household interviews. The difference in recall time lag was not longer than 1 week in most instances. Lastly, while results are consistent with those reported in previous SMC effectiveness studies in low and high resistance settings, the extent to which this study’s results can be generalized to other regions of South Sudan, or other settings where SMC is being introduced, is uncertain. Future deployment of SMC in other new geographies need to be informed by local evidence. That can be through the conduct of rapid assessment and pilot studies, as proposed in Malaria Consortium’s current implementation research efforts to generate vital evidence on the potential suitability of SMC in a range of settings with seasonal malaria transmission in East and Southern Africa.

## Conclusion

Overall, the results show high effectiveness of SMC using SPAQ in terms of preventing malaria disease during the high transmission season in children 3–59 month in Northern Bahr el-Ghazal. Despite the very promising effectiveness results, further evidence is required for a more accurate understanding of effectiveness and suitability of SMC in this setting. Results obtained in parallelly conducted resistance markers, chemoprevention efficacy and qualitative study components of the implementation research will contribute to a better understanding of the role of drug resistance in determining SMC effectiveness, efficacy and potential scalability in the region.

### Supplementary Information

**Additional file 1.** A graph representing the number of malaria-treated cases recorded per month in Aweil South during 2020. A table with the participant’s characteristics by county in Wave 2 survey. A table with the participants’ characteristics by county in Wave 3 survey. A table with the results of fully adjusted regression models (Model 3) for associations between SMC and caregiver-reported malaria outcomes among children aged 3–59 months using difference-in-differences analysis comparing Aweil South and Aweil West counties, June–November 2022. A table with the results of regression models for associations between SMC and caregiver-reported malaria outcomes among children aged 3–59 months using difference-in-differences analysis comparing Aweil South and Aweil West counties, June–November 2022.

## Data Availability

Processed data supporting the findings of this study are included in this published article and its supplementary information files. Original datasets generated and analysed during the current study are available from the corresponding author on reasonable request.
