# Supplementary material for: A quasi-experimental study to estimate effectiveness of seasonal malaria chemoprevention in Aweil South County in Northern Bahr El Ghazal, South Sudan
Source: Malar J. 2024 Jan 24;23:33. doi: 10.1186/s12936-024-04853-x (PMC10810022; doi:10.1186/s12936-024-04853-x)
Supplement: Supplementary file 1 — Additional file 1. A graph representing the number of malaria-treated cases recorded per month in Aweil South during 2020. A table with the participant’s characteristics by county in Wave 2 survey. A table with the participants’ characteristics by county in Wave 3 survey. A table with the results of fully adjusted regression models (Model 3) for associations between SMC and caregiver-reported malaria outcomes among children aged 3–59 months using difference-in-differences analysis comparing Aweil South and Aweil West counties, June–November 2022. A table with the results of regression models for associations between SMC and caregiver-reported malaria outcomes among children aged 3–59 months using difference-in-differences analysis comparing Aweil South and Aweil West counties, June–November 2022. [file 12936_2024_4853_MOESM1_ESM.docx]

# Additional file

**Figure S1**. **Malaria-treated cases recorded per month in Aweil South during 2020**. The peak in the number of recorded malaria-treated cases observed between July and November corresponds to the rainy season period of the region.


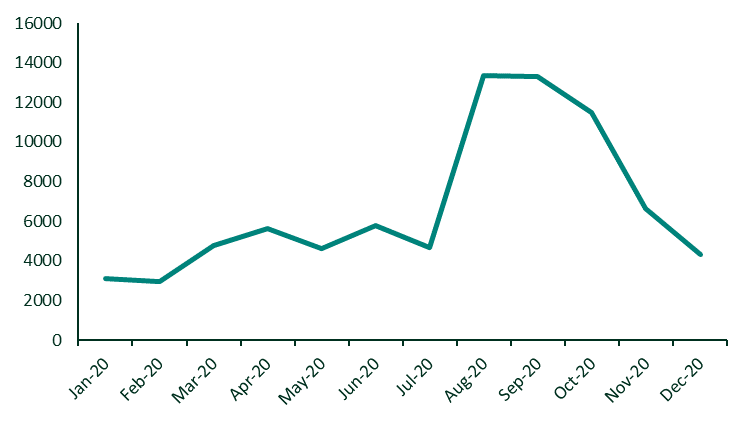


| **Table S1. Participants’ characteristics by county in Wave 2 survey** | | | | | | | | |  |
| --- | --- | --- | --- | --- | --- | --- | --- | --- | --- |
| **Variable** | | **Category** | **Aweil South (intervention)** | | | **Aweil West (Control)** | | |  |
|  |  |  | n | % | weighted % | n | % | weighted % | p |
| **Child** | **Fever** | Yes | 235 | 54.5% | 52.8% | 442 | 93.8% | 93.6% | 0.000 |
|  |  | No | 196 | 45.5% | 47.2% | 29 | 6.2% | 6.4% |  |
|  | **RDT-confirmed malaria** | Yes | 177 | 41.1% | 40.5% | 355 | 75.4% | 75.3% | 0.000 |
|  |  | No | 254 | 58.9% | 59.5% | 116 | 24.6% | 24.7% |  |
|  | **Sex** | Male | 216 | 50.1% | 54.0% | 240 | 51.0% | 51.5% | 0.557 |
|  |  | Female | 215 | 49.9% | 46.0% | 231 | 49.0% | 48.5% |  |
|  | **Age** | 3–12 months | 33 | 7.7% | 9.0% | 50 | 10.6% | 10.1% | 0.602 |
|  |  | 1 year | 60 | 13.9% | 14.0% | 74 | 15.7% | 16.1% |  |
|  |  | 2 years | 101 | 23.4% | 23.7% | 89 | 18.9% | 18.4% |  |
|  |  | 3 years | 112 | 26.0% | 24.3% | 107 | 22.7% | 21.4% |  |
|  |  | 4 years | 88 | 20.4% | 20.7% | 108 | 22.9% | 21.8% |  |
|  |  | 5 years | 37 | 8.6% | 8.3% | 43 | 9.1% | 12.2% |  |
| **Caregiver** | **Age** | Under 20 years | 58 | 13.5% | 10.2% | 65 | 13.8% | 21.5% | 0.283 |
|  |  | 20–29 years | 159 | 36.9% | 39.0% | 201 | 42.7% | 39.2% |  |
|  |  | 30–39 years | 165 | 38.3% | 38.6% | 147 | 31.2% | 27.3% |  |
|  |  | 40–49 years | 41 | 9.5% | 10.1% | 46 | 9.8% | 9.8% |  |
|  |  | 50 or 59 years | 8 | 1.9% | 2.1% | 12 | 2.5% | 2.3% |  |
|  | **Sex** | Male | 107 | 24.8% | 22.8% | 106 | 22.5% | 23.0% | 0.9799 |
|  |  | Female | 324 | 75.2% | 77.2% | 365 | 77.5% | 77.0% |  |
|  | **Partnership status** | Married/partnered | 408 | 94.7% | 95.1% | 413 | 87.7% | 82.4% | 0.0131 |
|  |  | Non-partnered | 23 | 5.3% | 4.9% | 58 | 12.3% | 17.6% |  |
|  | **Literacy** | Yes | 210 | 48.7% | 40.3% | 242 | 51.4% | 55.0% | 0.2694 |
|  |  | No | 221 | 51.3% | 59.7% | 229 | 48.6% | 45.0% |  |
|  | **Education** | None | 291 | 67.5% | 65.2% | 198 | 42.0% | 42.5% | 0.006 |
|  |  | Informal | 83 | 19.3% | 22.2% | 73 | 15.5% | 12.2% |  |
|  |  | Primary or above | 57 | 13.2% | 12.6% | 200 | 42.5% | 45.2% |  |
|  | **Occupation** | Non-employed | 72 | 16.7% | 11.9% | 61 | 13.0% | 13.2% | 0.356 |
|  |  | Unemployed | 33 | 7.7% | 8.3% | 33 | 7.0% | 7.9% |  |
|  |  | Agricultural | 314 | 72.9% | 77.1% | 311 | 66.0% | 67.4% |  |
|  |  | Unskilled manual work | 2 | 0.5% | 0.8% | 33 | 7.0% | 6.9% |  |
|  |  | Skilled/Service/professional | 10 | 2.3% | 1.9% | 33 | 7.0% | 4.6% |  |
| **Household** | **Net ownership** | Yes | 286 | 66.4% | 64.5% | 346 | 70.5% | 74.0% | 0.4851 |
|  |  | No | 145 | 33.6% | 35.5% | 145 | 29.5% | 26.0% |  |

| **Table S2. Participants’ characteristics by county in Wave 3 survey** | | | | | | | | |  |
| --- | --- | --- | --- | --- | --- | --- | --- | --- | --- |
| **Variable** | | **Category** | **Aweil South (intervention)** | | | **Aweil West (Control)** | | |  |
|  |  |  | n | % | weighted % | n | % | weighted % | p |
| **Child** | **Fever** | Yes | 248 | 54.5% | 53.1% | 405 | 88.6% | 88.3% | 0.0035 |
|  |  | No | 207 | 45.5% | 46.9% | 52 | 11.4% | 11.7% |  |
|  | **RDT-confirmed malaria** | Yes | 181 | 39.8% | 38.8% | 353 | 77.2% | 73.6% | 0.002 |
|  |  | No | 274 | 60.2% | 61.2% | 104 | 22.8% | 26.4% |  |
|  | **Sex** | Male | 227 | 49.9% | 51.0% | 216 | 47.3% | 45.6% | 0.1531 |
|  |  | Female | 228 | 50.1% | 49.0% | 241 | 52.7% | 54.4% |  |
|  | **Age** | 3–12 months | 12 | 2.6% | 4.1% | 34 | 7.4% | 8.8% | 0.524 |
|  |  | 1 year | 100 | 22.0% | 21.5% | 89 | 19.5% | 19.1% |  |
|  |  | 2 years | 117 | 25.7% | 23.4% | 108 | 23.6% | 23.7% |  |
|  |  | 3 years | 118 | 25.9% | 23.5% | 112 | 24.5% | 21.5% |  |
|  |  | 4 years | 86 | 18.9% | 21.7% | 83 | 18.2% | 18.2% |  |
|  |  | 5 years | 22 | 4.8% | 5.8% | 31 | 6.8% | 8.7% |  |
| **Caregiver** | **Age** | Under 20 years | 77 | 16.9% | 15.4% | 82 | 17.9% | 21.2% | 0.467 |
|  |  | 20–29 years | 212 | 46.6% | 48.8% | 201 | 44.0% | 39.5% |  |
|  |  | 30–39 years | 124 | 27.3% | 26.9% | 120 | 26.3% | 26.7% |  |
|  |  | 40–49 years | 35 | 7.7% | 7.4% | 31 | 6.8% | 7.5% |  |
|  |  | 50 or 59 years | 7 | 1.5% | 1.5% | 23 | 5.0% | 5.1% |  |
|  | **Sex** | Male | 106 | 23.3% | 21.2% | 98 | 21.4% | 25.8% | 0.6104 |
|  |  | Female | 349 | 76.7% | 78.8% | 359 | 78.6% | 74.2% |  |
|  | **Partnership status** | Married/partnered | 415 | 91.2% | 91.1% | 388 | 84.9% | 80.9% | 0.1419 |
|  |  | Non-partnered | 40 | 8.8% | 8.9% | 69 | 15.1% | 19.1% |  |
|  | **Literacy** | Yes | 232 | 51.0% | 46.8% | 222 | 48.6% | 47.2% | 0.9588 |
|  |  | No | 223 | 49.0% | 53.3% | 235 | 51.4% | 52.8% |  |
|  | **Education** | None | 304 | 66.8% | 63.5% | 199 | 43.5% | 45.7% | 0.077 |
|  |  | Informal | 93 | 20.4% | 21.5% | 78 | 17.1% | 14.8% |  |
|  |  | Primarhy or above | 58 | 12.7% | 15.0% | 180 | 39.4% | 39.5% |  |
|  | **Occupation** | Non-employed | 88 | 19.3% | 17.6% | 54 | 11.8% | 15.0% | 0.331 |
|  |  | Unemployed | 43 | 9.5% | 7.9% | 33 | 7.2% | 8.4% |  |
|  |  | Agricultural | 315 | 69.2% | 71.6% | 316 | 69.1% | 65.4% |  |
|  |  | Unskilled manual work | 0 | 0.0% | 0.0% | 24 | 5.3% | 3.7% |  |
|  |  | Skilled/Service/professional | 9 | 2.0% | 2.9% | 30 | 6.6% | 7.5% |  |
| **Household** | **Net ownsership** | Yes | 347 | 76.3% | 76.1% | 338 | 74.0% | 71.2% | 0.7422 |
|  |  | No | 108 | 23.7% | 23.9% | 119 | 26.0% | 28.8% |  |

| **Table S3. Coverage results in the second and fourth SMC cycles in Aweil South county, 2022** | | | | |
| --- | --- | --- | --- | --- |
| **Cycle** | **Target population** | **Received Day 1 dose (95% CI)** | **Received Day 1 dose as directly observed treatment^#^ (95% CI)** | **Received the full three-day course^#^ (95% CI)** |
| cycle 2 | 18,514 | 92.0 (83.8-95.8) | 98.3 (96.6-99.2) | 88.2 (77.2-94.3) |
| cycle 4 |  | 93.8 (85.3-97.5) | 96.2 (92.6-98.1) | 99.2 (97.3-99.7) |

^#^ as a proportion (%) of children who received Day 1 dose

| **Table S4. Results of fully adjusted regression models (Model 3) for associations between SMC and caregiver-reported malaria outcomes among children aged 3–59 months using difference-in-differences analysis comparing Aweil South and Aweil West counties, June–November 2022** | | | | | | |
| --- | --- | --- | --- | --- | --- | --- |
| **Variable** | | **Category** | **Caregiver-reported fever outcomes** | | **Caregiver-reported RDT-confirmed malaria outcomes** | |
|  |  |  | **OR (95% CI)** | **p** | **OR (95% CI)** | **p** |
| **SMC Effect** | **County** | Aweil South (intervention) | 0.23 (0.11 – 0.50) | <0.001 | 1.13 (0.53 – 2.42) | 0.743 |
|  | **Wave** | Wave 2 | 3.47 (2.38 – 5.08) | <0.001 | 3.27 (1.56 – 6.88) | 0.003 |
|  |  | Wave 3 | 1.34 (0.76 – 2.36) | 0.307 | 3.68 (1.88 – 7.20) | <0.001 |
|  | **Interaction** | County*Wave 2 | 0.22 (0.10 – 0.49) | <0.001 | 0.22 (0.08 – 0.62) | 0.005 |
|  |  | County*Wave 3 | 0.69 (0.25 – 1.96) | 0.477 | 0.19 (0.05 – 0.64) | 0.009 |
| **Child** | **Sex** | Male | 1.24 (0.99 – 1.54) | 0.055 | 1.08 (0.88 – 1.31) | 0.444 |
|  |  | Female | ref |  | ref |  |
|  | **Age** | 3–12 months | 1.01 (0.60 – 1.71) | 0.966 | 1.41 (0.76 – 2.62) | 0.261 |
|  |  | 1 year | 0.56 (0.36 – 0.89) | 0.016 | 0.92 (0.54 – 1.57) | 0.753 |
|  |  | 2 years | 0.95 (0.67 – 1.35) | 0.787 | 1.21 (0.94 – 1.56) | 0.130 |
|  |  | 3 years | ref |  | ref |  |
|  |  | 4 years | 0.87 (0.60 – 1.24) | 0.430 | 0.93 (0.74 – 1.17) | 0.527 |
|  |  | 5 years | 1.30 (0.79 – 2.13) | 0.295 | 1.02 (0.34 – 3.04) | 0.966 |
| **Household** | **Net ownership** | Yes | 0.88 (0.52 – 1.48) | 0.622 | 1.09 (0.70 – 1.68) | 0.696 |
|  |  | No | ref |  | ref |  |
|  | **Housing quality scale** | Continuous | 0.64 (0.40 – 1.03) | 0.063 | 0.80 (0.53 – 1.21) | 0.287 |

| **Table S5. Results of regression models for associations between SMC and caregiver-reported malaria outcomes among children aged 3–59 months using difference-in-differences analysis comparing Aweil South and Aweil West counties, June–November 2022** | | | | | | | | | | | | |  |
| --- | --- | --- | --- | --- | --- | --- | --- | --- | --- | --- | --- | --- | --- |
| **Model** | | | | **A: Including children in intervention county who did not receive full course of SMC in each cycle and children in control county who received Day 1 SPAQ in any cycle** | | | **B: Excluding children in intervention county who did not receive full course of SMC in each cycle and children in control county who received Day 1 SPAQ in the previous cycle** | | | **C: Excluding children in intervention county who did not receive full course of SMC in each cycle and children in control county who received Day 1 SPAQ in the previous cycle including Day 1 and Day 2 AQ** | | | |
| **Outcome** | **Model description** | **Variable** | **Category** | **Odds**  **ratio** | **95% CI** | **p** | **Odds**  **ratio** | **95% CI** | **p** | **Odds**  **ratio** | **95% CI** | **p** | |
| **Caregiver-reported fever outcomes** | **Model 1:** unadjusted model | County | Aweil South (intervention) | 0.27 | 0.10–0.70 | 0.008 | 0.27 | 0.11–0.69 | 0.008 | 0.27 | 10.4–69.0 | 0.008 | |
|  |  | Wave | Wave 2 | 2.78 | 1.77–4.37 | <0.001 | 3.91 | 2.41–6.36 | <0.001 | 3.91 | 2.41–6.36 | <0.001 | |
|  |  |  | Wave 3 | 1.44 | 0.77–2.67 | 0.246 | 1.33 | 0.70–2.53 | 0.375 | 1.33 | 0.70–2.53 | 0.375 | |
|  |  | Interaction | County*Wave 2 | 0.29 | 0.12–0.70 | 0.007 | 0.20 | 0.08–0.50 | 0.001 | 0.21 | 0.09–0.51 | 0.001 | |
|  |  |  | County*Wave 3 | 0.56 | 0.20–1.54 | 0.252 | 0.62 | 0.22–1.75 | 0.356 | 0.62 | 0.23–1.74 | 0.354 | |
|  | **Model 2:** adjusted for child age and sex | County | Aweil South (intervention) | 0.26 | 0.11–0.62 | 0.004 | 0.26 | 0.11–0.69 | 0.004 | 0.26 | 0.11–0.69 | 0.004 | |
|  |  | Wave | Wave 2 | 2.60 | 1.68–4.04 | <0.001 | 3.61 | 2.19–5.94 | <0.001 | 3.59 | 2.17–5.92 | <0.001 | |
|  |  |  | Wave 3 | 1.39 | 0.72–2.66 | 0.315 | 1.29 | 0.65–2.58 | 0.451 | 1.29 | 0.65–2.58 | 0.456 | |
|  |  | Interaction | County*Wave 2 | 0.29 | 0.12–0.70 | 0.007 | 0.21 | 0.09–0.50 | 0.001 | 0.22 | 0.09–0.52 | 0.001 | |
|  |  |  | County*Wave 3 | 0.59 | 0.22–1.59 | 0.285 | 0.64 | 0.22–1.80 | 0.39 | 0.65 | 0.23–1.80 | 0.392 | |
|  | **Model 3:** full model | County | Aweil South (intervention) | 0.18 | 0.11–0.62 | <0.001 | 0.18 | 0.09–0.39 | <0.001 | 0.18 | 0.09–0.38 | <0.001 | |
|  |  | Wave | Wave 2 | 2.40 | 1.68–4.04 | 0.001 | 3.34 | 2.27–4.69 | <0.001 | 3.36 | 2.42–4.69 | <0.001 | |
|  |  |  | Wave 3 | 1.36 | 0.72–2.66 | 0.260 | 1.27 | 0.72–2.26 | 0.402 | 1.2 | 0.72–2.31 | 0.381 | |
|  |  | Interaction | County*Wave 2 | 0.30 | 0.12–0.70 | 0.003 | 0.22 | 0.11–0.45 | <0.001 | 0.23 | 0.11–0.47 | <0.001 | |
|  |  |  | County*Wave 3 | 0.63 | 0.22–1.59 | 0.306 | 0.67 | 0.26–1.74 | 0.404 | 0.67 | 0.26–1.75 | 0.407 | |

| **Caregiver-reported RDT-confirmed malaria outcomes** | **Model 1:** unadjusted model | County | Aweil South (intervention) | 1.10 | 0.51–2.23 | 0.807 | 1.10 | 0.51–2.38 | 0.807 | 1.10 | 0.51–2.38 | 0.807 | |
| --- | --- | --- | --- | --- | --- | --- | --- | --- | --- | --- | --- | --- | --- |
|  |  | Wave | Wave 2 | 3.61 | 1.93–6.77 | <0.001 | 3.34 | 1.56–7.18 | 0.003 | 3.35 | 1.56–7.18 | 0.003 | |
|  |  |  | Wave 3 | 3.32 | 1.91–5.76 | <0.001 | 3.69 | 1.91–6.75 | <0.001 | 3.59 | 1.91–6.75 | <0.001 | |
|  |  | Interaction | County*Wave 2 | 0.21 | 0.08–0.55 | 0.003 | 0.22 | 0.07–0.66 | 0.008 | 0.23 | 0.08–0.69 | 0.001 | |
|  |  |  | County*Wave 3 | 0.21 | 0.06–0.62 | 0.006 | 0.19 | 0.06–0.62 | 0.007 | 0.19 | 0.06–0.61 | 0.006 | |
|  | **Model 2:** adjusted for child age and sex | County | Aweil South (intervention) | 1.10 | 0.51–2.38 | 0.803 | 1.10 | 0.51–2.38 | 0.800 | 1.10 | 0.51–2.38 | 0.802 | |
|  |  | Wave | Wave 2 | 3.61 | 2.03–6.43 | <0.001 | 3.34 | 1.66–6.71 | 0.001 | 3.33 | 1.64–6.74 | 0.001 | |
|  |  |  | Wave 3 | 3.31 | 1.91–5.73 | <0.001 | 3.59 | 1.86–6.91 | <0.001 | 3.58 | 1.86–6.89 | <0.001 | |
|  |  | Interaction | County*Wave 2 | 0.20 | 0.08–0.54 | 0.002 | 0.22 | 0.08–0.63 | 0.006 | 0.23 | 0.08–0.66 | 0.008 | |
|  |  |  | County*Wave 3 | 0.21 | 0.07–0.63 | 0.006 | 0.19 | 0.06–0.63 | 0.008 | 0.19 | 0.06–0.62 | 0.007 | |
|  | **Model 3:** full model | County | Aweil South (intervention) | 0.94 | 0.45–1.93 | 0.860 | 0.96 | 0.46–1.98 | 0.901 | 0.95 | 0.44–2.00 | 0.890 | |
|  |  | Wave | Wave 2 | 3.39 | 1.79–6.43 | <0.001 | 3.24 | 1.52–6.89 | 0.003 | 3.26 | 1.50–7.04 | 0.004 | |
|  |  |  | Wave 3 | 3.39 | 1.89–6.08 | <0.001 | 3.85 | 1.95–7.62 | <0.001 | 3.88 | 1.95–7.71 | <0.001 | |
|  |  | Interaction | County*Wave 2 | 0.18 | 0.07–0.49 | 0.001 | 0.20 | 0.07–0.57 | 0.004 | 0.21 | 0.07–0.60 | 0.005 | |
|  |  |  | County*Wave 3 | 0.18 | 0.06–0.54 | 0.003 | 0.16 | 0.05–0.55 | 0.004 | 0.16 | 0.05–0.53 | 0.004 | |
|  | | | | | | | | | | | | |  |
